# Fine-scale spatial genetic structure and dispersal among Italian smooth newt populations in a rural landscape

**DOI:** 10.1038/s41598-023-47265-8

**Published:** 2023-11-15

**Authors:** Vincenzo Buono, Alessandra Maria Bissattini, Francesca Davoli, Chiara Mengoni, Nadia Mucci, Leonardo Vignoli

**Affiliations:** 1 Department of Biology and Biotechnologies “Charles Darwin”, Sapienza University of Rome, 00185 Rome, Italy; 2 Unit for Conservation, Management and Sustainable Use of Marine Aquatic Resources (BIO-CIT), Department for the Monitoring and Protection of the Environment and for Biodiversity Conservation, Italian Institute for Environmental Protection and Research (ISPRA), Ozzano Dell’Emilia, 40064 Bologna, Italy; 3 Unit for Conservation Genetics (BIO-CGE), Department for the Monitoring and Protection of the Environment and for Biodiversity Conservation, Italian Institute for Environmental Protection and Research (ISPRA), Ozzano Dell’Emilia, 40064 Bologna, Italy; 4 Department of Science, Roma Tre University, 00146 Rome, Italy

**Keywords:** Population genetics, Herpetology, Conservation biology

## Abstract

Amphibians are particularly sensitive to habitat loss and fragmentation caused by the intensification and modernization of farming occurring in the second half of the twentieth century in the Mediterranean basin. However, artificial water bodies, associated with traditional husbandry, proved to be important surrogate for amphibian feeding and reproduction. Here, multilocus genotypes were used to investigate the spatial population structure of *Lissotriton vulgaris meridionalis* and the role of drinking troughs in supporting viable breeding populations within a rural landscape interested by traditional husbandry and agriculture. Our genetic analysis highlighted the conservation value and the potential stepping-stone function of artificial aquatic sites in the dispersal of the species and for the gene flow maintenance. Indeed, populations of drinking troughs show allelic richness and heterozygosity levels comparable to those from natural ponds and there is no great evidence of genetic bottlenecks. A complex system of artificial aquatic sites and few natural wetlands was identified sustaining a well-structured network of demes highly interconnected with themselves and natural aquatic sites. The conservation of the identified genetic clusters may be useful to prevent further population declines and future loss of genetic diversity within the study area characterized by scarce natural wetlands that frequently dried because of agricultural practices and strong seasonality. Site-specific protection measures are needed to contrast the progressive disappearance of drinking troughs observed in the last years in Italy because of the abandonment of traditional farming practices in favour of modern agriculture and intensive farming.

## Introduction

The European landscape is the result of the dynamic interaction between people and their natural environments^1^. This is particularly true for the Mediterranean basin, a biodiversity hotspot, in which the current mosaic of agricultural lands, traditional pastures, evergreen woodlands and maquis habitats, is the result of anthropogenic activities over millennia^2,3^. Particularly, traditional agricultural systems, derived from well-integrated and sustainable management practices, being spatially heterogeneous, have supported a high level of biodiversity^1,4^. However, in the second half of the twentieth century, the intensification of modern agricultural practices has increased habitat loss and fragmentation, spatial homogenization, and a reduction in biodiversity^5^. In general, amphibians are particularly sensitive to habitat destruction and fragmentation that have a negative impact at a demographic and genetic level^6^ with habitat connectivity playing a key role in their viability at the regional scale^7^. Indeed, agriculture and urban development activities may create barriers to amphibian dispersal^8,9^ resulting in isolated subpopulations more subject to increased risk of inbreeding and local extinction^10,11^, and/or less likely to be recolonized by dispersers from neighbouring subpopulations^12^. In an isolated population, a decrease in the immigration rate of individuals from other populations (i.e. gene flow) will inevitably produce a genetic diversity loss^6^. This happens when population size is reduced^13^ and the isolation among populations is so strong that inbreeding and genetic drift are no longer counterbalanced by gene flow^14^. These negative effects are further exacerbated by the fact that amphibians, due to their habitat specificity, strict ecophysiological requirements^15^, low dispersal abilities, and philopatry^16^, do not behave as single, infinitely large panmictic population^17^ but as patchy demes representing unique genetic entities despite that geographic proximity. In addition, due to their biphasic life cycle in aquatic and terrestrial habitats, amphibians are vulnerable to the alteration of both environments and to any factors reducing population connectivity^18^. Despite human activities negatively impact amphibian populations, many studies in the Mediterranean highlighted the importance of artificial water bodies, associated with traditional agriculture and cattle watering, for amphibian feeding and reproduction^19–22^. This suggests that such artificial facilities, often connected to natural water bodies, may represent important surrogate breeding habitats for amphibians^19,20,23^. Indeed, the ability of amphibians to colonise artificial aquatic sites (e.g. tanks, drinking troughs and reservoirs) has been well documented worldwide^19,24,25^. Moreover, they may act as stopovers during dispersal events, thereby facilitating amphibians’ movements by reducing effective inter-aquatic systems distances^26,27^. However, there are no studies on their role and importance as for the population genetic conservation and for stepping-stone function in the dispersal process and gene flow maintenance.

**Figure 1:**
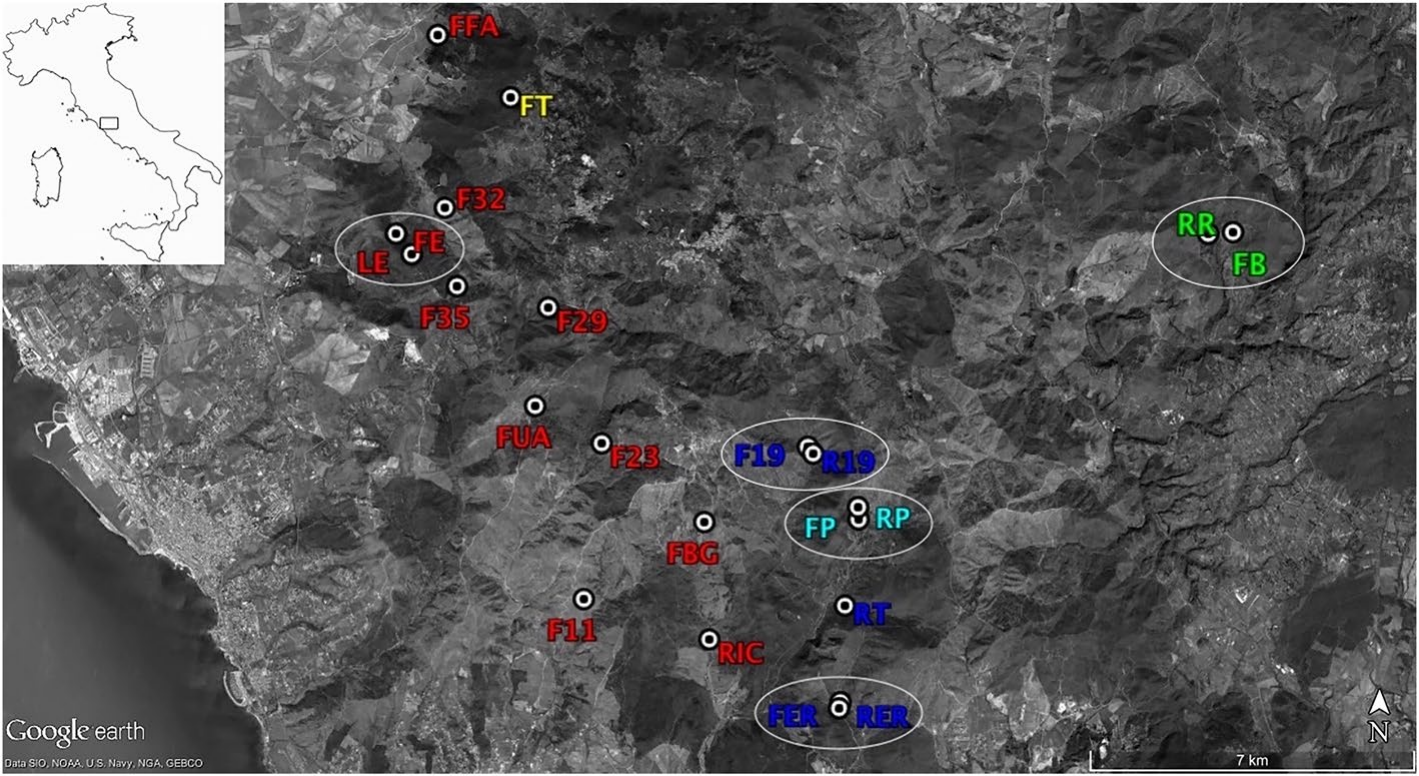
Topographic map of Tolfa Mountains, with localities for 21 *Lissotriton vulgaris meridionalis* populations sampled for this study. Each coloured ID corresponds to the assigned cluster. White circles identify the five artificial-natural site pairs.

With increasing anthropogenic habitat modification, it is particularly important to understand the genetic consequences of changes in landscape composition and configuration^28^. In this context, microsatellite markers have proved to be sensitive indicators of change in dispersal rates and suitable tools for addressing questions related to population subdivision and interconnection even at fine scale^29^. Studies on the individual movements and the spatial scale of dispersal among subpopulations are needed in addition to basic demographic research for improving our knowledge of amphibian population dynamics and sustainability, and to assist landscape management and conservation planning^12,30^. However, species genetic management is often overlooked in conservation programmes due to lack of resources or expertise^31^. However, the determination of genetic relationships among populations may be particularly useful to detect areas of high conservation priority, dispersal corridors, population declines and individuals and/or populations fundamental for augmentation/translocation or reintroduction programs^32^. Here, we described the spatial population structure of the Italian smooth newt *Lissotriton vulgaris meridionalis* (Linnaeus 1758) in a rural landscape of Central Italy (Latium) within a patchy system interested by infrastructural and agricultural development (Fig. 1). We aimed at investigating the role of drinking troughs used for cattle watering and traditional agricultural practices in supporting viable amphibian breeding populations and if and how they are connected to other natural (i.e. brooks) and/or artificial aquatic systems. We selected the sub-endemic Italian smooth newt as a model species since it is widely distributed in the study area (Latium, Central Italy)^33^ where it is known to successfully colonize both natural and artificial aquatic sites^34^. Specifically, multilocus genotypes (microsatellite-based protocol) were used to (i) evaluate genetic diversity within and among artificial and natural breeding sites; (ii) infer population structure in the study area; (iii) detect migration and gene flow among demes; (iv) establish whether drinking troughs may provide alternative and/or complementary aquatic habitats for amphibian conservation in rural landscapes.

## Results

### Within-population patterns of genetic diversity

Genotypic data were obtained from 464 individuals, excluding six samples that had more than 20% missing genotypes. One locus (Lm_013) was also excluded from data analyses because of a large amount of missing data, due to an amplification failure.

Estimated null allele frequencies were not significant, indicating the absence of null alleles, large allele dropout and false homozygotes at any locus in all populations.

Microsatellite markers were highly polymorphic across populations sampled, except for LVG-210 and LVG-388, which were monomorphic in 3 and 9 populations, respectively (Table S1, Supplementary material). Overall, allele numbers per locus ranged from 3 (LVG-388) to 46 (Lm_488 and Lm_749). Across all loci, expected heterozygosity (*H*e) ranged from 0.048 to 0.955, and observed heterozygosity (*H*o) from 0.048 to 1.00 (Table S1, Supplementary material).

All but three loci (Lm_521, Tv3Ca19, Lm_488) conformed to Hardy–Weinberg equilibrium after Bonferroni correction, with ten populations showing a slight deviation at one locus (Table S1, Supplementary material). We assumed that processes causing disequilibrium were specific to these populations, and thus decided to include the three loci in subsequent analyses.

The number of alleles ranged from 4.5 (FP) to 10.6 (FER) in artificial sites and from 5.4 (RP) to 9.7 (RER) in natural water bodies. Allelic richness varied from 4.4 (FP) to 8.4 (FER) in drinking troughs and from 5.0 (RP) to 8.1 (RIC) in natural sites (Table 1).

**Table 1:** Details of study populations.

Pop code	N	Na	Ar	Pa	Ho	He	Fis (P value)
FB (1)	17	6.000 (0.915)	5.888	0.455 (0.207)	0.613 (0.085)	0.617 (0.078)	0.037 (0.281)
RR (1)	33	7.818 (1.340)	6.760	0.364 (0.364)	0.629 (0.083)	0.681 (0.070)	0.092 (0.002)
FT	20	4.727 (0.764)	4.552	0.182 (0.122)	0.515 (0.102)	0.507 (0.086)	0.012 (0.507)
FFA	20	6.909 (1.443)	6.573	0.091 (0.091)	0.591 (0.098)	0.630 (0.100)	0.088 (0.023)
F32	20	7.364 (1.429)	6.924	0.091 (0.091)	0.646 (0.089)	0.654 (0.082)	0.037 (0.199)
FE (2)	20	7.727 (1.613)	7.164	0.091 (0.091)	0.559 (0.097)	0.619 (0.093)	0.122 (0.001)*
LE (2)	20	8.091 (1.729)	7.614	0.091 (0.091)	0.670 (0.099)	0.662 (0.087)	0.014 (0.562)
F35	20	7.909 (1.575)	7.463	0.000 (0.000)	0.625 (0.075)	0.656 (0.069)	0.074 (0.038)
F29	20	5.818 (1.069)	5.608	0.000 (0.000)	0.527 (0.077)	0.583 (0.079)	0.122 (0.004)
FUA	20	7.818 (1.747)	7.386	0.000 (0.000)	0.555 (0.089)	0.621 (0.090)	0.132 (0.0001)*
F23	20	8.455 (1.755)	7.854	0.273 (0.141)	0.623 (0.096)	0.637 (0.099)	0.048 (0.119)
F11	20	7.545 (1.592)	7.026	0.000 (0.000)	0.541 (0.086)	0.600 (0.091)	0.123 (0.002)
RIC	17	8.273 (1.722)	8.120	0.182 (0.122)	0.602 (0.099)	0.647 (0.093)	0.100 (0.008)
FBG	20	7.364 (1.403)	7.008	0.000 (0.000)	0.627 (0.096)	0.636 (0.094)	0.040 (0.160)
F19 (3)	20	6.909 (1.609)	6.537	0.091 (0.091)	0.573 (0.103)	0.565 (0.102)	0.012 (0.409)
R19 (3)	20	7.818 (1.710)	7.364	0.000 (0.000)	0.568 (0.110)	0.607 (0.101)	0.089 (0.013)
RP (4)	21	5.364 (1.106)	4.983	0.000 (0.000)	0.550 (0.106)	0.547 (0.103)	0.019 (0.357)
FP (4)	20	4.545 (0.608)	4.447	0.091 (0.091)	0.573 (0.091)	0.566 (0.081)	0.014 (0.421)
RT	30	9.545 (2.121)	7.861	0.364 (0.203)	0.602 (0.094)	0.652 (0.097)	0.095 (0.001)*
FER (5)	34	10.636 (2.394)	8.455	0.364 (0.279)	0.668 (0.095)	0.659 (0.095)	0.002 (0.556)
RER (5)	32	9.727 (2.253)	8.013	0.182 (0.182)	0.627 (0.105)	0.654 (0.097)	0.056 (0.034)

Number of smooth newts genotyped (*N*), allele number (*N*a), allelic richness (*A*r), private alleles (*P*a), mean observed (*H*_O_) and expected (*H*_E_) heterozygosities for the eleven microsatellite loci, and *F*_IS_ with *P* value (*significantly greater than zero in 3 of the 21 populations, using a randomization test with 10.100 randomizations) for each of the sampled *Lissotriton vulgaris meridionalis* breeding populations. The numbers in brackets identify natural/artificial pairs.

A few populations showed slightly lower levels of diversity (FT, RP, FP). Expected (*H*e) heterozygosity varied from 0.507 (FT) to 0.659 (FER) in artificial sites, and from 0.547 (RP) to 0.681 (RR) in natural sites. Observed heterozygosity (*H*o) ranged from 0.515 (FT) to 0.668 (FER) in artificial sites and from 0.550 (RP) to 0.670 (LE) in natural sites (Table 1).

Overall, *F*_IS_ values ranged from 0.002 to 0.132 with no indication of homozygote excess (or heterozygosity deficit), except for three populations (FE, FUA and RT) showing *F*_IS_ estimates significantly greater than zero after Bonferroni correction (Table 1).

No linkage disequilibrium was observed between any pair of loci, indicating independence of the eleven genetic markers.

Heterozygosity did not vary between artificial and natural sites (F_1,19_ = 1.537, p = 0.230). No significant differences were observed in *H*e values within each site pair represented by a natural and an artificial site (N = 5, Z = 1.214, p = 0.225).

### Among-population patterns of genetic diversity

Pairwise *F*_ST_ values ranged from 0.001 (FER-RER) to 0.239 (FT-FB) with an average *F*_ST_ = 0.066. *F*_ST_ values were significantly higher than zero in 202 of 210 cases after sequential Bonferroni correction for multiple comparisons. *F*_ST_ was not significant in three out five natural/artificial site pairs (FE-LE, F19-R19, RER-FER). Overall, low to moderate levels of population differentiation were observed except for FT showing the highest *F*_ST_ values (Table S2, Supplementary material).

The number of migrants (*N*m) between populations, calculated directly from the *F*_ST_ values (Table S2, Supplementary material; above diagonal), ranged from 0.797 individuals (FT-FP) to 249.75 individuals per generation (FER-RER). The lowest *N*m estimates calculated from pairwise *F*_ST_ values ranged from 0.797 to 1.845 for FT comparisons.

**Figure 2:**
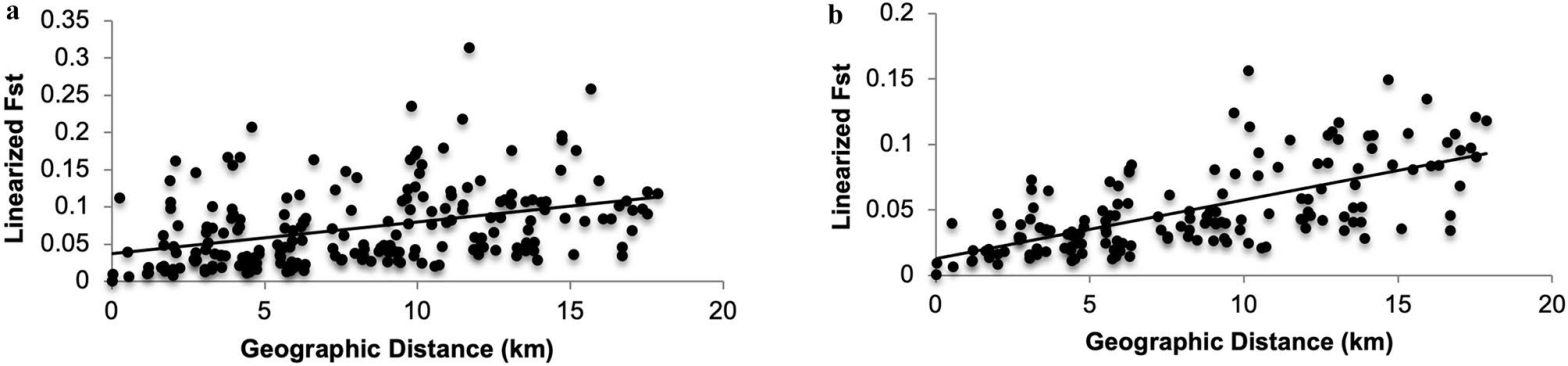
Relationship between pairwise geographic and genetic distances (linearized *F*_ST_) across *Lissotriton vulgaris meridionalis* populations. Mantel tests reveal a significant correlation in both cases. (**a**) All sampled populations; (**b**) populations with exclusion of potential outliers.

Genetic distances (*F*_ST_) among pairs of all populations were significantly correlated with the geographical distance between localities (Mantel test, *P* = 0.013). However, geographical distance explained less than 14% of the genetic variation (*r*^2^ = 0.137) in the study area (Fig. 2a). The subsequent test for Isolation by Distance (IBD) without outliers (FT, FP, and RP) revealed a higher significant correlation with geographical distance (*P* = 0.001) explaining about 43% of the genetic variation (*r*^2^ = 0.428) (Fig. 2b).

The pairwise relatedness analysis showed a slight presence of close relatives within our populations. Based on Lynch & Ritland’s estimator, we identified the presence of second- and third-order of relationships (in FT and FP populations, and in FB, F29 and RP populations, respectively). No close relatives were present within the five site pairs (see Table S3, Supplementary material).

**Figure 3:**
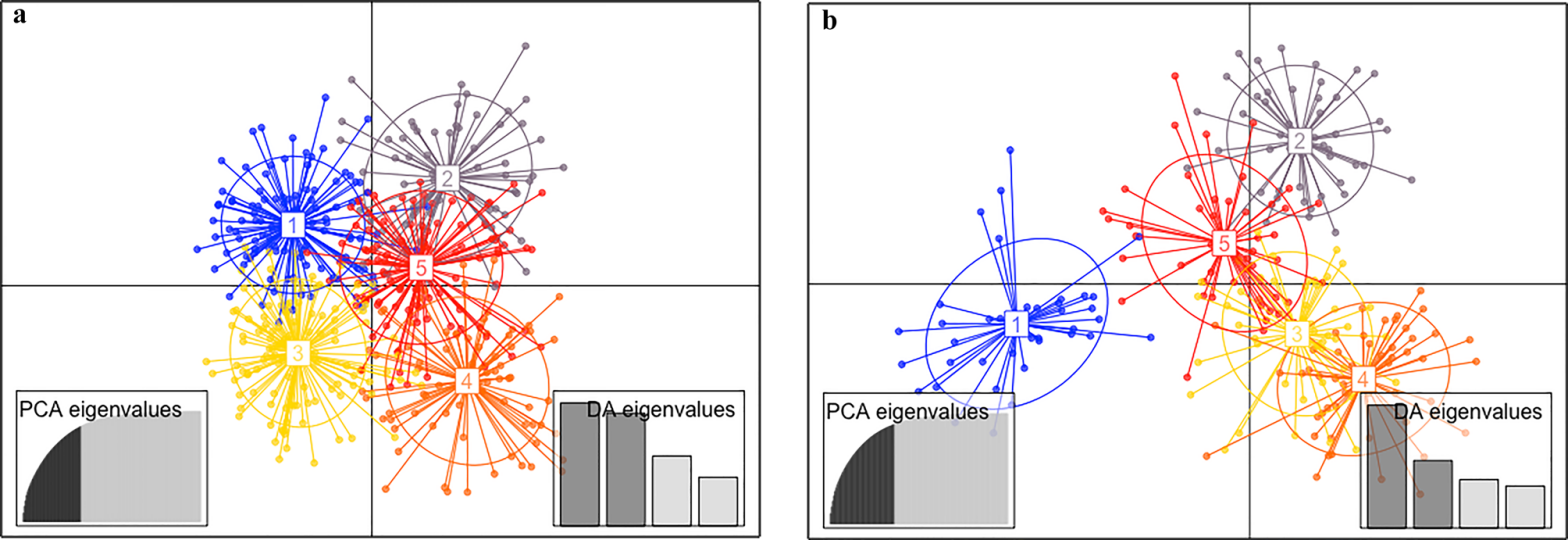
Population genetic structure by multivariate clustering methods (Discriminant Analysis of Principal Components, DAPC): (**a**) Discriminant analysis of the genetic variation in Italian smooth newts from 21 breeding sites; (**b**) analysis of the genetic differentiation among the five artificial-natural site pairs.

The discriminant analysis of principal components (DAPC) identified 5 clusters. The resulting scatterplot showed evidence of fair separation among clusters (Fig. 3a), with some individuals that did not clearly belong to a distinct group. Considering the membership probabilities of each individual for the different groups, FB-RR and FT populations formed two distinct clusters (clusters 2 and 4, respectively), whereas cluster 1, 3 and 5 consisted mainly of individuals from populations of north-western area (FFA, F32, FE, LE, F35, F29, FUA, F23), south-eastern area (FER, RER, RT) and eastern area (FP, RP), respectively. Individuals from the remaining populations seemed to be admixed and had no distinct division, otherwise belonging to clusters 1, 3 and 5.

DAPC revealed a pronounced genetic differentiation among the five artificial-natural site pairs (Fig. 3b), highlighting that within each pair populations from drinking troughs and natural water bodies are genetically interconnected and represent a unique deme (Fig. 3b).

### Bayesian estimates of genetic structure

The model-based clustering method suggested that the model with K = 5 (where K is the number of population genetic clusters) was substantially better than alternative models.

**Figure 4:**
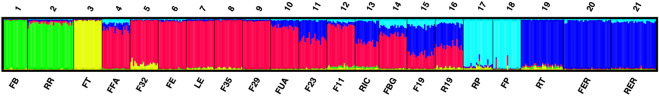
Population structure inferred by Bayesian assignment of 464 individuals of *Lissotriton vulgaris meridionalis* performed by STRUCTURE software. Italian smooth newt populations in Tolfa Mountains can be assigned to five clusters of populations (*K* = 5).

Five clusters were identified with FT was split from the other populations. FB-RR and FP-RP formed two clusters on their own. All the other populations clustered in two distinct groups corresponding to north-western and south-eastern geographical areas (Fig. 4).

**Figure 5:**
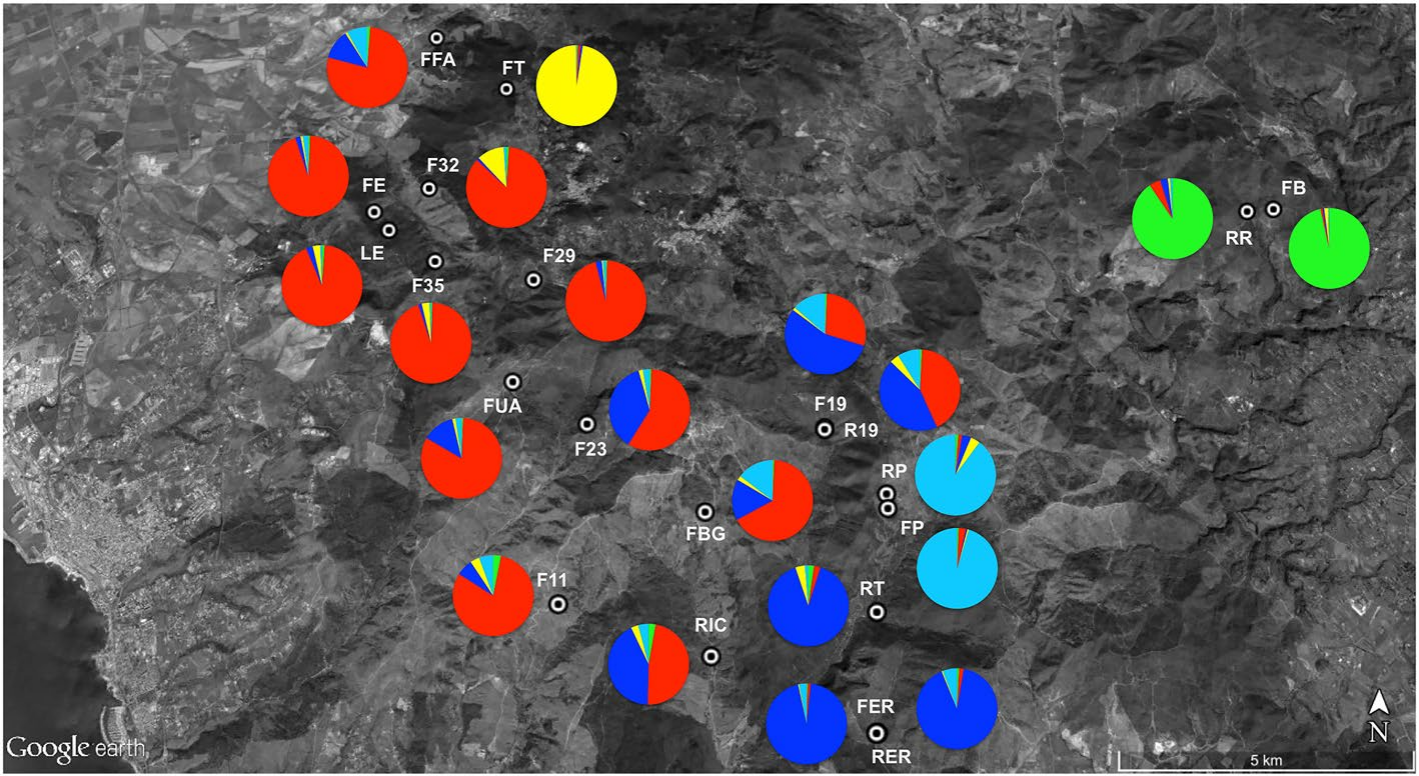
Topographic map of Tolfa Mountains, with localities for 21 *Lissotriton vulgaris meridionalis* populations sampled for this study. Pie charts describe the percentage membership to each cluster for *K* = 5.

Mean membership coefficients for all individuals into the north-western and south-eastern demes was generally high, with a mean of 0.796 (SD = 0.160) and 0.751 (SD = 0.235), respectively. A few populations (F23, RIC, FBG, F19 and R19), located on the borders of these two clusters, showed membership coefficients less than 70% and thus may be considered as admixed demes (Figs. 4, 5).

The hierarchical analysis of molecular variance (AMOVA) confirmed the genetic structure detected by clustering methods, with 86.73% of genetic variation resided within populations, 7.89% distributed among populations within clusters, and 5.38% of the variance possibly explained by differentiation among the five clusters (Table 2). Although the proportion of genetic variation accountable at higher levels is small (5.38%), all fixation indices were statistically significant. Results were similar even if we excluded admixed populations (F23, RIC, FBG, F19 and R19; Table 2).

**Table 2:** Results of hierarchical Amova comparing genetic variation within breeding populations, among breeding populations within the five clusters, and among clusters.

Source of variation	d.f	Sum of square	Percentage of variation	*P* value
Including admixed populations				
Within populations	464	1524.000	86.73	< 0.001*
Among populations within clusters	20	1781.911	7.89	< 0.001*
Among clusters	4	143.065	5.38	< 0.001*
Excluding admixed populations				
Within populations	367	1205.000	85.78	< 0.001*
Among populations within clusters	15	1402.708	7.73	< 0.001*
Among clusters	4	144.574	6.49	< 0.001*

Significance was tested against a null distribution of 10,000 random permutations. The test was performed twice, including and excluding admixed populations. Significant *P* values are indicated with an asterisk.

### Bottleneck detection

The hypothesis of a recent bottleneck was tested with infinite allele model (IAM), stepwise mutation model (SMM) and two-phase model (TPM). We observed the occurrence of a bottleneck event in 7 out of 21 populations under IAM model (RR, FFA, FE, FBG, FP, RT, and RER; Table 3). Significant heterozygote excess was detected in RT population (p = 0.032) also under SMM (Table 3). Moreover, under the more relaxed hypothesis of a certain proportion of mutations encompassing more than a single repeat unit (TPM), the results suggested that two populations had faced a recent bottleneck (FFA and FBG; Table 3). On the contrary, no evidence of heterozygote deficit (i.e. a sign of expansion) was detected.

**Table 3:** Wilcoxon test for 21 *Lissotriton vulgaris meridionalis* populations under three mutation models: the infinite allele model (IAM), the stepwise mutation model (SMM) and the two-phase model (TPM, with 90% single-step mutations and 10% multiple-step mutations).

Pop	IAM	TPM	SMM
FB	0.130859	0.492188	0.232422
RR	**0.016113**	0.053711	0.413086
FT	0.275391	0.769531	0.130859
FFA	**0.001953**	**0.001953**	0.496094
F32	0.278320	0.101563	0.965820
FE	**0.012207**	0.898438	0.147461
LE	0.067383	0.123047	0.831055
F35	0.067383	0.519531	0.083008
F29	0.067383	0.365234	0.320313
FUA	0.053711	0.240234	0.637695
F23	0.083984	0.322266	0.130859
F11	0.320313	0.965820	0.206055
RIC	0.240234	0.413086	0.764648
FBG	**0.009766**	**0.032227**	0.375000
F19	0.083984	0.232422	0.769531
R19	0.160156	0.375000	1.0
RP	0.232422	0.275391	1.0
FP	**0.009766**	0.083984	0.845703
RT	**0.032227**	0.232422	**0.032227**
FER	0.147461	0.577148	0.053711
RER	**0.009766**	0.105469	0.845703

Significant *p-*values are indicated in bold attesting the occurrence of a recent bottleneck event.

Overall, no significant shift in distribution was detected in our study populations that remained in a normal L-shape, as revealed by the allele frequency distribution test (Fig. S1, Supplementary material).

### Recent migration rates

Few pairwise comparisons between localities (5%) detected recent migrations. In 20 instances, the migration rate was greater than 0.04 indicating the occurrence of substantial contemporary gene flow. In 12 cases, migration rates were > 0.1 suggesting that more than 10% of the population is composed by recent immigrants. The highest level of gene flow was mostly detected in populations that were geographically closed and belonging to the same genetic cluster (FB-RR; FP-RP; FER-RER; Table S4, Supplementary material).

**Figure 6:**
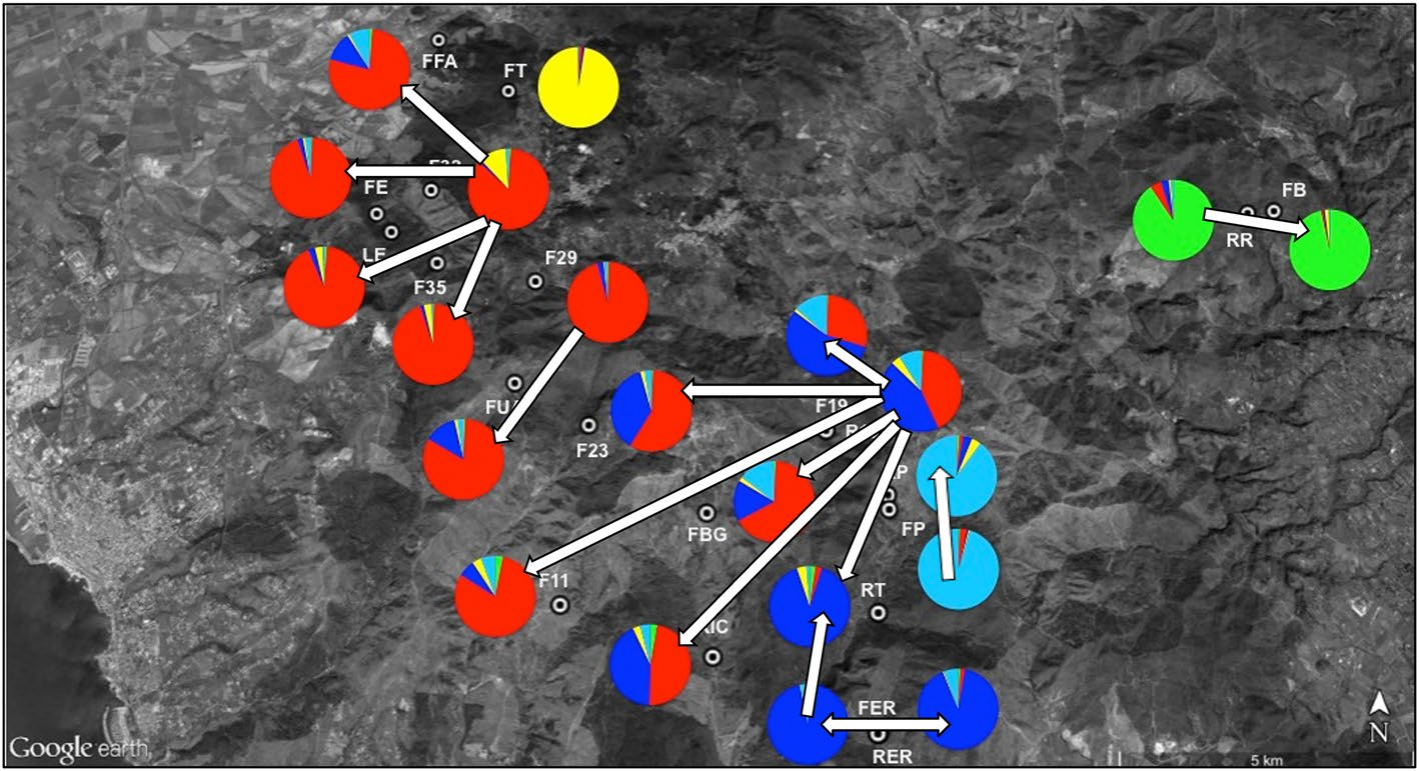
Topographic map of Tolfa Mountains, with localities for 21 *Lissotriton vulgaris meridionalis* populations sampled for this study. Pie charts describe the percentage membership to each cluster for *K* = 5. The white arrows indicate the direction of gene flow among newt populations at the study area.

These incidences of migration included emigrations from only seven source populations (RR to FB; F32 to FFA, FE, LE, F35, FUA, F23, F11; F29 to FUA; R19 to FUA, F23, F11, RIC, FBG, F19, RT; FP to RP; FER to RER, RT; RER to FER; Fig. 6).

## Discussion

To our knowledge, this is the first study to examine the distribution of genetic variation and the spatial genetic structure of Italian smooth newt (*Lissotriton vulgaris meridionalis*) within a rural area of Central Italy (Latium).

During the last century, this rural area was subjected to a reduction of natural water bodies and habitat fragmentation due to human activities. In a previous study, we showed how traditional livestock farming, by building drinking troughs for cattle watering, provided suitable sites for amphibian reproduction, representing a surrogate for the lost natural ones^19^. Here, we pointed out that their capillary distribution seems to mitigate habitat fragmentation by increasing the number of demes, supporting gene flow among them, and thus improving the overall network of aquatic systems.

Populations showed a similar genetic diversity with no differences between natural and artificial wetlands, comparable to other urodeles and temperate amphibians^35^. Moreover, newts from drinking troughs had a genetic diversity (i.e. heterozygosity) comparable to those from the closest brook. As the level of genetic diversity reflects population’s adaptive and evolutionary potential^36^, this finding demonstrates the importance of artificial habitats for the persistence of amphibians especially where original habitats have been degraded or lost^20^.

Smooth newts are highly philopatric, with short dispersal ability^37^, resulting in large genetic differentiation among populations that may facilitate local adaptation or cause genetic drift^38^. To date, for *L. vulgaris meridionalis*, genetic data are only available from^17^, who found moderate to high differentiation levels among close populations. In our study, genetic differentiation among populations was moderate (average *F*_ST_ = 0.066), thus indicating the existence of a network of populations connected by dispersing individuals^39^. This suggests that drinking troughs contributed to sustain gene flow, decreasing local genetic divergence, and mitigating genetic drift and population inbreeding^40^. Our findings are in agreement with previous fine-scale studies of urodeles employing microsatellite markers^12,29,41–43^.

Some populations showed higher pairwise *F*_ST_-values not consistent with the geographic distribution (i.e. FT, FP, and RP). This may be due to the relatively longer timespan since isolation and the higher habitat fragmentation characterising FT and FP-RP demes. It is likely that, in the past, the study area consisted of a unique unfragmented woody landscape with many natural aquatic systems used by newts for reproduction. In the 1950s, wide-scale human impact caused habitat loss and fragmentation isolating some demes (i.e. FT and FP-RP) in less favourable areas. The transition forests-arable lands or grazed-cultivated lands, and the construction of roads may have removed suitable terrestrial shelters and artificial breeding sites used for reproduction^44^, affecting the connectivity of local populations, dispersal, and genetic diversity^36,45^. Our suppositions are in agreement with previous studies documenting the negative effects of habitat fragmentation on local genetic variability and connectedness in amphibians^9,46–48^.

We also found a correlation between genetic and geographical distance measures. Isolation by distance is expected to shape genetic variation, even on small spatial scales, leading to populations more differentiated from each other with increasing distance^49,50^. Indeed, we found a significant IBD pattern between populations separated by a maximum distance of 16.7 km (FFA-FER) and 17.8 km (FB-FE). We thus confirm the limited dispersal ability of *L. vulgaris meridionalis* even at small spatial scale^43^, whose average dispersal distance has been estimated to be 400–500 m^51^. However, this moderate correlation coefficient detected suggests that further processes other than distance may have structured populations’ genetic composition^48^. Indeed, dispersal capability is known to be not only determined by species’ intrinsic biology but also by landscape features and habitat quality^52,53^. In this context, it is likely that natural and artificial water bodies have increased survival probability and facilitated juvenile dispersal and adult breeding migration^43^. Drinking troughs, in particular, seem to act as stepping-stone system connecting distant natural breeding sites and building a network able to face potential isolation.

Five statistically significant population clusters with a moderate genetic differentiation have been identified. Gene flow seems to be extensive among populations within each cluster determining quite genetically homogeneous assemblages, with a genetic variation attributable to within-populations differences. Moreover, the five artificial-natural site pairs were genetically interconnected representing each a unique nearly panmictic deme. Such a positive association confirms the potential of drinking troughs to contrast drift and local loss of genetic diversity providing a network of permanent aquatic sources used for breeding and migration^9,54^.

Three clusters had a very similar cohesiveness concordant with the geographic distribution evidencing a significant positive relationship between genetic differentiation and geographical distance^55^. This probably reflects the counterbalance of different effects currently ongoing or acted in the past. On one hand, natural limits to dispersal and population-level processes may have affected site fidelity and low vagility increasing genetic distance^56^; on the other, gene flow among pristine habitats, once widespread in the study area, may have reduced genetic differentiation. Two clusters drifted apart probably because of landscape features alone or in combination with topographical differences. Indeed, both natural barriers and human-induced environmental changes affect occasional migration events among distant populations in *L. vulgaris meridionalis* hindering gene flow and resulting in a strong genetic structure^43^.

Five populations showed evidence of mixed genetic ancestry. Such admixed populations are expected in systems where geographical structure exists but there are no barriers to dispersal^41^. Tolfa Mountains conform to this landscape scenario being characterized by large patches of natural habitats interspersed with areas subject to traditional agricultural and husbandry activities without absolute geographic barriers limiting individual exchange among populations^57^.

Signals of current migration were found within clusters but not among populations from different sites. Interestingly, migration was unidirectional and influenced by the hydrology of the site (permanent vs. temporary) rather than its typology (natural vs. artificial). Surprisingly, not only natural but also artificial permanent aquatic sites acted as sources of individuals. The observed configuration of water network and the connectivity among drinking troughs, and between drinking troughs and natural aquatic systems, suggests the existence of a metapopulation with a source-sink configuration, in which recolonization following local extinctions, and natural restocking after demographic contractions are likely to occur^58^.

We found evidence of a slight occurrence of bottleneck events (RT, FFA and FBG). Amphibian populations are known to fluctuate from year to year with a demographic and connectivity instability^42^ resulting in a genetic bottleneck effect at local scale. This also suggests that populations did not recently experience severe effects of natural and anthropogenic pressures, or such pressures were mitigated by the positive impact of the observed configuration of water network. RT, a brook population subject to periodic drought, seems to have experienced a recent bottleneck probably counterbalanced by individuals migrating from neighbouring natural and artificial sites. FFA and FBG drinking troughs populations probably suffered inadequate management practices, associated with mechanical and chemical cleaning activities and water extraction^19^. Such operations may cause early desiccation and metamorphosis failure of larvae, with massive mortalities every year and consequent repercussions on the occurrence and abundance of species at local scale^20^ and a decline in genetic variation^13^.

Our study revealed that a complex system of artificial permanent aquatic sites and few natural wetlands can sustain a well-structured network of amphibian demes that seem to be quite connected. The current landscape matrix suggests that the observed genetic structure may not reflect present-day gene flow but an intermediate situation between the historic scenario after the 1950s^57^ and the current landscape. Drinking trough populations probably derived from individuals in dispersal from neighbouring natural sites subject to a relatively fast drift known to affect small and isolated populations^59^. Since 15–20 generations are needed for signals of genetic effects to be observable^60^, the genetic structure inferred is more likely to represent a snapshot of past landscape configuration and composition rather than contemporary ones^61^.

Our study highlights the significant role played by the Maremmana cow in *L. vulgaris meridionalis* conservation. The Maremmana cow seems to act as an ecosystem engineer (i) directly by structuring landscape features (i.e. increasing habitat heterogeneity) and (ii) indirectly by providing suitable artificial sites crucial for newt genetic diversity maintenance. We emphasize how drinking troughs may represent an interesting tool for promoting individual dispersal and gene flow, reinforcing metapopulation patterns observed in the study area. Intervention measures are timely to prevent the progressive abandonment of drinking troughs observed in the last 50 years in Italy due to expansion of modern and intensive farming^62,63^. At the local scale, conserving and managing the existing natural sites as well as incentivization of traditional farming accompanied by drinking trough restoration, preservation and proper management will increase the availability of water supplies to both the Maremmana cow and newts, preventing further population declines within the study area. Particularly, the correction of trap effects, invasive species eradication, cleaning practices respectful of newt phenology, intervention to avoid disrepair and permeability loss^19,20,23^ will help to preserve habitat suitability maximizing the effectiveness of drinking troughs as stepping-stones. At the regional scale, habitat suitability of both natural and artificial water bodies could be improved by increasing site connectivity^64^. Indeed, the co-occurrence of aquatic sites differing in sizes and other features (e.g. vegetation cover, feeding resources) in the landscape is known to favour species’ dispersal reinforcing their metapopulations^64^.

Our conclusions suggest that amphibian protection and social rural and cultural values preservation can be simultaneously achieved combining conservation biologists’ efforts and local livelihood needs^19^.

## Methods

### Study area

The study area falls within the Maremma district, extended throughout Latium and Tuscany regions. Fieldwork was carried out in the Comprensorio Tolfetano-Cerite-Manziate (Province of Rome, Latium Region, Central Italy; elevations between 150 and 450 m a.s.l.) a Special Protection Area (SPA, IT6030005), spanning 70,000 ha, included in the European Ecological Natura 2000 network. It is characterized by a high biodiversity value representing one of the highest wilderness areas in Italy despite the proximity to several main cities and its rural profile interested by traditional agriculture and husbandry. A mosaic of pastures, cultivated lands, and woodlands characterizes the vegetation landscape, mainly consisting of riverine woods, wide areas covered by bushy pastures and mesophilous forests^19,65^.

In this area, natural wetlands are scarce and are frequently dried due to agricultural practices and their strong seasonality. Indeed, they are characterized by a short hydroperiod, especially in summer, because of high temperatures and irregular rainfalls. However, artificial drinking troughs built by farmers after World War II to facilitate livestock watering (cattle, horses, and donkeys), are particularly widespread. Drinking troughs are small, shallow (20–60 cm depth) habitats, with rectangular shape and vertical walls, often in connection with natural permanent or semi-permanent aquatic systems^19,66^. Such artificial aquatic sites are particularly used by the Maremmana cow (*Bos primigenius taurus*), a robust and frugal native breed well adapted to difficult environments living freely in the marshes and maquis of the south coastal area of Italy for more than 1500 years^67,68^.

### Population sampling

All the potential breeding sites (natural and artificial) were identified through orthophotos in Google Earth Pro software (version 7.3.1.4507). Overall, 7 natural aquatic sites (one pond and six brooks, named LE, RR, RIC, R19, RP, RT, RER) and 51 functioning drinking troughs were detected within an area covering approximately 18,000 ha. We focused on all 7 natural sites and on 14 drinking troughs (labelled with F in Table 1; highest elevation of 398 m a.s.l. at F29 site) characterized by the presence of *L. vulgaris meridionalis* and a distribution across the study area aimed at minimizing spatial autocorrelation (i.e. space distance of at least 1 km; Fig. 1).

Finally, we selected a study model consisting of five artificial-natural water body pairs, each consisting of a drinking trough built in the immediate surrounding (< 500 m) of a natural brook/pond (FB-RR; RP-FP; FE-LE; F19-R19; RER-FER; see Fig. 1). This approach has been adopted since natural and artificial sites are interconnected, and artificial sites most likely originated from the colonization of individuals from natural sites.

Study sites were sampled during the breeding season of the species (March–May) in 2015–2016 years. Since cohorts can vary in genetic composition, we avoided sampling the same site at different times during the year^41^. We surveyed drinking troughs by dip netting^69^, whereas newts were visually located and captured by hand or dip nets in natural sites. We collected 470 tissue samples by tail-clipping^70^ considering at least 20 adult individuals from each aquatic site useful for the analysis. Samples were stored in ethanol 95% at room temperature until genetic analysis.

Tissue samples were obtained with no anaesthetic and by careful handling of animals. After this procedure, individuals were temporarily housed in tanks filled with water for approximately 30 min to verify their return to normal activity and released at the same point of capture. No individuals died or were otherwise harmed during samplings. This study was conducted according to the ethical scientific guidelines described by the American Society of Ichthyologists and Herpetologists (ASIH), The Herpetological League (HL) and the Society for the Study of Amphibians and Reptiles (SSAR).

Experimental protocols were approved by Italian Ministry of the Environment that gave LV the permit [0001255/PNM] to manipulate amphibians and collect tissue samples.

All methods are reported in accordance with ARRIVE guidelines.

### DNA extraction and microsatellite amplification

Total genomic DNA was extracted using DNeasy Blood and Tissue Kit (QIAGEN) and QIAcube robotic workstation, then amplified at 12 microsatellite loci (STRs) optimized for this species^71^. Three multiplex PCRs (M1 included Lm_521, LVG-210/EU568357, LVG-388/EU568352 and LVG-542/EU568354; M2 included Lm_488, Lm_632, Th09 and Tv3Ca9; M3 included Lm_013, Lm_749, LVG-398/EU568356 and Tv3Ca19) were performed to save time and reagents. PCRs were carried out using Qiagen Multiplex PCR Kit in an 8 μL mix composed of 3.50 μL HotStarTaq Master Mix, 0.70 μL Qsol, 1.50 μL DNA, 0.20 μL each primer (10 μM), brought to volume with H_2_O. Amplification conditions for all loci included an initial denaturing at 95 °C for 15 min; 40 cycles of denaturing at 94 °C for 30 s, annealing temperature of 55 °C for 1.30 min, and 72 °C extension for 1 min; and a final 10 min extension at 72 °C. Two replicates for each sample at each locus were executed to confirm the genotypes.

Forward primers for each locus were 5’-labelled with a fluorescent dye for detection (Applied Biosystems). PCR products were electrophoresed with internal size standard (GeneScan 1200 LIZ, Applied Biosystems) in an ABI Prism 3130XL sequencer and allele sizes were scored using the software GeneMapper v.5.0 (Applied Biosystems).

### Intra and inter-population patterns of genetic diversity

The presence of null alleles was checked by Micro‐Checker v.2.2.3^72^.

Genetic diversity indices (number of alleles, effective number of alleles and number of private alleles) were calculated for each population by GenAlex v.6.5^73,74^. Allelic richness was computed in Fstat 2.9.3^75^. Arlequin v.3.5.2^76^ was used to estimate observed (*H*_O_) and expected (*H*_E_) heterozygosity and to test genotypic frequencies at each population and locus for statistically significant deviations from Hardy–Weinberg equilibrium (HWE). We used a Monte Carlo approximation of the Fisher’s exact test^77^ and a standard Bonferroni correction for multiple comparisons (adjusted P-value < 0.001, for a table-wide significance of α = 0.05). The Markov chain algorithm was run for 100,000 steps following 10,000 dememorization steps. The pairwise probability of linkage disequilibrium was estimated using a Fisher’s exact test implemented in Genepop v.4.2^78^ with 10,000 steps following 1000 dememorization steps.

Analysis of variance (ANOVA) was used to reveal differences in expected heterozygosity as a function of site type (F, artificial; R, natural). Moreover, the Wilcoxon matched-pairs test was performed to compare heterozygosity values within each site pair represented by a natural and an artificial site. Statistical tests were performed using STATISTICA software (version 8.0 for Windows) with alpha set at 5%.

Fstat 2.9.3 was used to perform a global test of overall population differentiation (not assuming HWE within populations)^75^. Pairwise significance tests for *F*_ST_^79^ were performed by permutation and resampling of multilocus genotypes among pairs of samples with 10,100 randomizations allowed for a table-wide significance at the 5% nominal level after standard Bonferroni corrections (adjusted P value = 0.001).

The degree of gene flow among demes, in terms of success of immigrants, was determined using *N*m estimates, derived from the fixation coefficient *F*_ST_^80^.

Pairwise population linearized *F*_ST_ values^81^ were correlated with geographical distances to test for patterns of spatial subdivision and isolation by distance (IBD) using a Mantel test with 1000 randomizations (implemented in GenAlex v.6.5). We performed two tests for IBD separately, on (i) all populations, and on (ii) populations with the exclusion of potential outliers (i.e. populations showing higher genetic distance despite the similar geographic distance in respect to the others).

Pairwise relatedness analysis was carried out to assess the presence of close relatives in our populations. We performed the analysis (implemented in GenAlex v.6.5) for each population and for the five artificial-natural site pairs using Lynch & Ritland’s^82^ LR estimator by 2 to give a maximum value of 1 and minimum of − 1.

A discriminant analysis of principal components (DAPC) was performed to assess the genetic divergence among populations and provide a description of the genetic clustering based on the largest between‐group and smallest within‐group variances in allele coefficients (R package “adegenet”)^83,84^. We used “find.clusters” function to identify the optimal number of clusters. *k*-means algorithm was run comparing different clustering solutions using Bayesian Information Criterion (BIC) values with increasing values of *k*. The optimal number of clusters was selected corresponding to the lowest BIC. Then, we determined the number of PCs to retain considering the cumulated variance explained by the eigenvalues of the PCA. After the cross-validation step, DAPC was performed with the retention of 60 PCs (> 80% of variance explained) and four discriminant functions. A further DAPC was conducted to verify genetic differentiation within the five artificial-natural site pairs previously identified, with the retention of 60 PCs and four discriminant functions.

### Bayesian estimates of genetic structure

We performed Bayesian clustering analysis to characterize the population genetic structure of aquatic systems across the study area and assess the geographical scale of population differentiation using STRUCTURE v2.3.4^85^. We ran 15 independent simulations of 1,000,000 iterations (following a burn-in period of 100,000) for each value of *K*^85^ between 1 and 21. The admixture model was applied assuming gene flow among populations and allowing for correlated allele frequencies across populations. We performed two different models: (i) the default mode for STRUCTURE that uses only genetic information to infer population structure, and (ii) the LOCPRIOR model that uses sampling locations as prior information to assist the clustering.

Appropriate *K* was selected based on the natural log of Pr(X|K) values and the ∆*K* criterion^86^ provided by STRUCTURE HARVESTER v.0.6.94^87^. Replicate runs were averaged using CLUMPP v.1.1^88^ and plotted in DISTRUCT v.1.1^89^ to generate graphical displays representing the mean membership of each individual over the replicate runs.

Considering the genetic clusters inferred from the Bayesian analyses as panmictic demes, an Analysis of Molecular Variance (AMOVA)^90^, implemented in Arlequin v.3.5.2^76^, was performed on *F*_ST_ values to examine the distribution of genetic variation at three hierarchical levels: within populations, among populations within demes, and among demes. We assigned each population to a cluster based on its largest proportion of membership and then examined hierarchical genetic distribution considering only populations with a mean membership ≥ 0.7 and excluding admixed populations. The significance of the variance components was tested using 10,000 permutations.

### Bottleneck detection

The software BOTTLENECK v1.2.02^91^ was used to test for evidence of recent bottlenecks in each population. The main assumption of this test is that allele frequency distribution results from an equilibrium between mutation and genetic drift^92^. In non-bottlenecked populations, the expected heterozygosity at mutation-drift equilibrium *(H*eq) equals the measured Hardy–Weinberg equilibrium heterozygosity *(H*e). Instead, in case of recent bottlenecks, a correlative reduction of allelic diversity and heterozygosity at polymorphic loci occurs and the mutation-drift equilibrium is temporarily disrupted: *H*e will be significantly greater than *H*eq, calculated from the number of alleles sampled^92,93^. The calculation of *H*eq depends on the mutation model considered analysing the loci^92^. In the present study, *H*eq values were calculated under three mutation models: the infinite allele model (IAM)^94^, the Stepwise Mutation Model (SMM)^95^ and a combination of those two extreme hypotheses, the two-phase model (TPM, with 90% single-step mutations and 10% multiple-step mutations)^96^. Significant deviations in observed heterozygosity across loci for each population were detected using a two-tailed Wilcoxon sign-rank test^97^ that allows the detection of the heterozygosity excess due to the faster loss of low frequency alleles and thus determines whether a population has been recently bottlenecked. Moreover, a qualitative descriptor of allele frequency distribution was used to infer bottlenecks. This method compares allele frequency distribution to the distribution expected at mutation-drift equilibrium (l-shaped distribution), considering that population bottlenecks cause a characteristic mode-shift distortion in the distribution of allele frequencies at selectively neutral loci^97^.

For fewer than 20 loci (i.e. 11 loci in our study), the Wilcoxon’s test is the most appropriate and powerful^91^. Since the IAM is mainly recommended for allozyme data and microsatellites are often described as being closer to the single step mutation model^91^ or to the Two-Phase Mutation model^96^, we may consider the results obtained under SMM and TPM more reliable.

### Recent migration rates

We estimated recent migration rates between localities using the program BAYESASS 3.0^98^, which employs a Bayesian MCMC approach to identify migrants or recent descendants of migrants based on transient linkage disequilibrium among multilocus genotypes from different source populations. This program was run following the recommendation of^99^ concerning convergence issues. We performed 10 separate runs, with different seed numbers, using 10^7^ iterations, with a burn-in of 10^6^ and a sampling frequency of 1000. This run length was sufficient for the posterior probability to achieve convergence. The MCMC mixing parameters were set to 0.50, 0.80 and 0.25, for allele frequency, level of inbreeding and migration rate, respectively. These delta values resulted in acceptance ratios between 40 and 60% to maximize log-likelihood values^98^. Migration rates were averaged over the 10 independent runs and compared to average migration rates of randomly permuted data set, generated to assess significance. Estimated migration rates were considered significant when the 95% confidence interval (CI) did not overlap with the 95% CI of the randomly permuted data.

### Supplementary Information

Supplementary Information.

## Data Availability

All data generated or analysed during this study are included in this published article and its supplementary information file.
