# Supplementary material for: Fine-scale spatial genetic structure and dispersal among Italian smooth newt populations in a rural landscape
Source: Sci Rep. 2023 Nov 15;13:19956. doi: 10.1038/s41598-023-47265-8 (PMC10651844; doi:10.1038/s41598-023-47265-8)

Supplementary materials

Table S1 Genetic variation at 11 microsatellite loci in populations of *Lissotriton vulgaris meridionalis*. For each breeding site, *N* equals the number of individuals genotyped and from which the observed (H_O) and expected (H_E) heterozygosities were estimated. Heterozygosities in bold indicate populations that do not conform to Hardy–Weinberg expectations for that particular locus (Bonferroni corrected *P*-value < 0.001 for table-wide significance level of α = 0.05)

		**LVG-210**		**LVG-388**		**LVG-542**		**Lm_521**		**Lm_488**		**Lm_632**		**Th09**		**Tv3Ca9**		**LVG-398**		**Lm_749**		**Tv3Ca19**	
**Pop Code**	***N***	***Ho***	***He***	***Ho***	***He***	***Ho***	***He***	***Ho***	***He***	***Ho***	***He***	***Ho***	***He***	***Ho***	***He***	***Ho***	***He***	***Ho***	***He***	***Ho***	***He***	***Ho***	***He***
**FB**	17	0.471	0.383	Monomorphic		0.412	0.447	0.941	0.872	0.941	0.891	0.941	0.831	0.471	0.651	0.647	0.679	0.647	0.731	0.706	0.777	0.563	0.730
**RR**	33	0.333	0.436	0.091	0.144	0.437	0.687	0.939	0.910	0.818	0.861	0.939	0.880	0.788	0.812	0.484	0.523	0.606	0.750	0.879	0.877	0.606	0.731
**FT**	20	Monomorphic		0.150	0.142	0.400	0.344	0.900	0.841	0.750	0.746	0.500	0.495	0.900	0.844	0.250	0.296	0.650	0.583	0.950	0.822	**0.211**	**0.612**
**FFA**	20	Monomorphic		Monomorphic		0.450	0.581	**0.600**	**0.928**	0.950	0.904	0.800	0.814	0.800	0.886	0.600	0.596	0.650	0.724	0.900	0.887	0.750	0.788
**F32**	20	0.150	0.142	0.105	0.193	0.600	0.556	0.800	0.938	0.950	0.905	0.950	0.905	0.800	0.783	0.750	0.612	0.600	0.760	0.900	0.883	0.500	0.695
**FE**	20	0.100	0.097	0.050	0.050	0.250	0.592	**0.750**	**0.937**	0.850	0.882	0.850	0.905	0.850	0.858	0.450	0.483	0.450	0.582	0.950	0.881	0.600	0.717
**LE**	20	0.050	0.050	0.250	0.232	0.750	0.658	0.700	0.931	1.000	0.946	0.950	0.883	1.000	0.853	0.750	0.595	0.450	0.712	1.000	0.938	0.471	0.677
**F35**	20	0.300	0.328	0.250	0.312	0.421	0.459	0.850	0.912	0.800	0.885	0.900	0.883	0.850	0.909	0.500	0.558	0.650	0.622	0.900	0.883	0.450	0.653
**F29**	20	0.150	0.142	0.150	0.142	0.500	0.431	**0.550**	**0.868**	0.650	0.729	0.850	0.832	0.900	0.851	0.550	0.636	0.450	0.622	0.750	0.847	0.300	0.481
**FUA**	20	0.250	0.224	0.050	0.050	0.400	0.467	**0.550**	**0.933**	0.950	0.950	0.800	0.844	0.900	0.924	0.450	0.568	0.450	0.554	0.900	0.912	0.400	0.579
**F23**	20	Monomorphic		0.050	0.050	0.650	0.674	0.750	0.924	**0.800**	**0.945**	0.850	0.879	0.900	0.900	0.550	0.573	0.800	0.663	0.900	0.899	0.600	0.676
**F11**	20	0.150	0.142	0.050	0.050	0.400	0.560	**0.650**	**0.935**	0.800	0.892	0.750	0.797	0.850	0.906	0.750	0.585	0.450	0.559	0.800	0.888	0.300	0.451
**RIC**	17	0.059	0.059	0.176	0.169	0.353	0.590	**0.588**	**0.916**	1.000	0.955	1.000	0.909	0.941	0.922	0.412	0.522	0.588	0.750	0.882	0.941	0.625	0.605
**FBG**	20	0.100	0.097	Monomorphic		0.450	0.612	0.900	0.900	0.900	0.927	0.800	0.836	0.850	0.882	0.550	0.650	0.700	0.687	0.850	0.895	0.800	0.691
**F19**	20	0.050	0.050	Monomorphic		0.450	0.440	0.700	0.945	0.950	0.912	0.900	0.863	0.850	0.810	0.500	0.385	0.600	0.622	0.950	0.922	0.350	0.426
**R19**	20	0.150	0.145	Monomorphic		0.350	0.501	0.700	0.949	0.900	0.918	1.000	0.900	0.950	0.914	0.350	0.541	0.750	0.755	0.900	0.906	0.200	0.314
**RP**	21	0.048	0.048	Monomorphic		0.619	0.617	0.905	0.886	0.810	0.832	0.857	0.756	0.762	0.843	0.429	0.494	0.619	0.738	0.905	0.855	0.095	0.094
**FP**	20	0.100	0.097	Monomorphic		0.800	0.691	0.550	0.783	0.900	0.777	0.900	0.771	0.550	0.769	0.700	0.579	0.650	0.685	0.750	0.676	0.400	0.559
**RT**	30	0.133	0.128	Monomorphic		0.667	0.572	**0.567**	**0.899**	0.867	0.938	0.867	0.886	0.933	0.873	0.400	0.563	0.767	0.790	0.867	0.936	0.552	0.712
**FER**	34	0.176	0.214	0.059	0.058	0.647	0.713	0.853	0.924	0.909	0.952	0.941	0.904	0.824	0.879	0.382	0.335	0.853	0.770	0.912	0.928	0.794	0.687
**RER**	32	0.125	0.119	Monomorphic		0.469	0.550	0.906	0.910	1.000	0.936	0.906	0.906	0.875	0.899	0.469	0.493	0.812	0.802	0.906	0.945	**0.433**	**0.745**

Table S2 Pairwise F_{ST} (below diagonal) and *N*m (above diagonal) estimates for 21 *Lissotriton vulgaris meridionalis* populations genotyped at 11 microsatellite loci. Values in bold were not statistically significant in exact tests of differentiation, assuming a table-wide significance level of $P<0.05$ (adjusted $P = 0.001$)

Pop Code	**FB**	**RR**	**FT**	**FFA**	**F32**	**FE**	**LE**	**F35**	**F29**	**FUA**	**F23**	**F11**	**RIC**	**FBG**	**F19**	**R19**	**RP**	**FP**	**RT**	**FER**	**RER**
**FB**		6.310	0.966	2.070	2.319	2.117	2.756	2.462	1.670	2.301	2.331	1.853	2.344	2.278	1.598	2.203	1.469	1.718	2.420	2.141	2.403
**RR**	0.038		1.422	2.616	2.980	2.572	3.654	2.990	2.584	2.952	3.055	3.093	3.615	2.922	2.014	3.221	2.323	2.247	3.030	2.339	2.906
**FT**	0.206	0.149		1.547	1.713	1.501	1.607	1.497	1.205	1.530	1.695	1.396	1.845	1.431	1.062	1.526	1.147	0.797	1.421	1.279	1.318
**FFA**	0.108	0.087	0.139		7.310	12.143	10.350	5.052	2.956	7.225	9.852	3.789	8.837	5.816	4.244	5.814	2.332	2.280	6.976	5.440	7.296
**F32**	0.097	0.077	0.127	0.033		23.468	13.177	12.429	3.419	7.788	10.088	5.167	12.062	6.286	4.000	6.173	2.551	2.062	5.484	4.835	6.127
**FE**	0.106	0.089	0.143	0.020	**0.011**		38.767	18.608	3.867	15.107	17.211	5.534	11.503	6.228	5.861	7.194	2.593	1.979	5.964	4.775	6.147
**LE**	0.083	0.064	0.135	0.024	0.019	**0.006**		22.328	4.834	11.948	20.005	5.736	10.084	9.367	6.311	8.901	3.164	2.159	6.954	5.546	7.257
**F35**	0.092	0.077	0.143	0.047	0.020	**0.013**	0.011		5.298	19.056	10.092	5.599	9.479	7.208	5.101	8.454	2.589	1.973	5.325	4.300	5.152
**F29**	0.130	0.088	0.172	0.078	0.068	0.061	0.049	0.045		6.482	3.815	3.167	4.069	3.484	3.100	4.555	2.614	1.798	3.098	2.672	3.271
**FUA**	0.098	0.078	0.140	0.033	0.031	0.016	0.021	**0.013**	0.037		13.308	7.902	13.439	11.421	9.440	17.201	3.517	2.037	6.681	5.129	6.369
**F23**	0.097	0.076	0.129	0.025	0.024	0.014	0.012	0.024	0.062	0.018		6.805	19.874	8.762	8.121	13.020	2.790	2.235	10.910	8.437	8.865
**F11**	0.119	0.075	0.152	0.062	0.046	0.043	0.042	0.043	0.073	0.031	0.035		8.583	5.833	5.484	12.673	3.420	2.157	5.485	3.645	4.593
**RIC**	0.096	0.065	0.119	0.028	0.020	0.021	0.024	0.026	0.058	0.018	**0.012**	0.028		13.717	7.464	22.657	3.605	2.784	15.761	12.364	15.742
**FBG**	0.099	0.079	0.149	0.041	0.038	0.039	0.026	0.034	0.067	0.021	0.028	0.041	0.018		6.452	9.176	3.504	2.487	7.069	5.920	6.735
**F19**	0.135	0.110	0.191	0.056	0.059	0.041	0.038	0.047	0.075	0.026	0.030	0.044	0.032	0.037		26.258	4.047	1.844	7.337	6.034	7.054
**R19**	0.102	0.072	0.141	0.041	0.039	0.034	0.027	0.029	0.052	0.014	0.019	0.019	**0.011**	0.027	**0.009**		5.171	2.349	13.695	7.648	10.132
**RP**	0.145	0.097	0.179	0.097	0.089	0.088	0.073	0.088	0.087	0.066	0.082	0.068	0.065	0.067	0.058	0.046		2.235	3.324	2.996	3.426
**FP**	0.127	0.100	0.239	0.099	0.108	0.112	0.104	0.112	0.122	0.109	0.101	0.104	0.082	0.091	0.119	0.096	0.101		2.546	2.560	2.958
**RT**	0.094	0.076	0.150	0.035	0.044	0.040	0.035	0.045	0.075	0.036	0.022	0.044	0.016	0.034	0.033	0.018	0.070	0.089		15.218	30.325
**FER**	0.105	0.097	0.163	0.044	0.049	0.050	0.043	0.055	0.086	0.046	0.029	0.064	0.020	0.041	0.040	0.032	0.077	0.089	0.016		249.750
**RER**	0.094	0.079	0.159	0.033	0.039	0.039	0.033	0.046	0.071	0.038	0.027	0.052	0.016	0.036	0.034	0.024	0.068	0.078	0.008	**0.001**	

Table S3 Estimates of pairwise relatedness within 21 smooth newt populations and five site pairs using Lynch & Ritland's method (LRM). Values indicating second- and third-order of relationships are pointed out with two and one asterisk, respectively.

**Single Populations**	
**ID**	**LRM**
**FB**	0.180*
**RR**	0.098
**FT**	0.293**
**FFA**	0.071
**F32**	0.075
**FE**	0.050
**LE**	0.037
**F35**	0.054
**F29**	0.136*
**FUA**	0.045
**F23**	0.033
**F11**	0.062
**RIC**	0.031
**FBG**	0.071
**F19**	0.055
**R19**	0.020
**FP**	0.251**
**RP**	0.169*
**RT**	0.026
**FER**	0.042
**RER**	0.029
**Population Pairs**	
**ID**	**LRM**
**FB/RR**	0.071
**FE/LE**	0.024
**F19/R19**	0.033
**FP/RP**	0.074
**FER/RER**	0.035

Table S4 Bayesian estimates of recent migration rates among smooth newt populations using the program BAYESASS 3.0 (Wilson and Rannala 2003). Source populations are listed across the top row; populations receiving immigrants are listed in the left-hand column. Each cell contains the mean migration rate and confidence interval (in brackets) for a pair of populations. Self-assignment rates are listed along the diagonal in italics. Most estimates of interpopulation migration are low, and do not differ significantly from values expected in cases of insufficient signal in the data (noninformative CI: 0-0.037). 20 cases show detectable migration rates, with an estimated mean larger 0.037 (in bold): these cases are immigrations from seven populations (RR, F32, F29, R19, FP, FER and RER), acting as a source for exchange among populations at this scale

Pop ID	FB	RR	FT	FFA	F32	FE	LE	F35	F29	FUA	F23	F11	RIC	FBG	F19	R19	RP	FP	RT	FER	RER
**FB**	*0.675 (0.659-0.692)*	**0.161 (0.111-0.211)**	0.009 (0-0.025)	0.009 (0-0.025)	0.008 (0-0.024)	0.009 (0-0.025)	0.009 (0-0.025)	0.008 (0-0.024)	0.009 (0-0.025)	0.009 (0-0.025)	0.009 (0-0.025)	0.009 (0-0.025)	0.009 (0-0.025)	0.009 (0-0.025)	0.009 (0-0.025)	0.009 (0-0.025)	0.009 (0-0.025)	0.009 (0-0.025)	0.009 (0-0.024)	0.009 (0-0.025)	0.009 (0-0.026)
**RR**	0.006 (0-0.018)	*0.850 (0.802-0.898)*	0.009 (0-0.024)	0.006 (0-0.017)	0.009 (0-0.024)	0.007 (0-0.020)	0.006 (0-0.018)	0.006 (0-0.018)	0.008 (0-0.025)	0.006 (0-0.018)	0.006 (0-0.018)	0.007 (0-0.019)	0.006 (0-0.017)	0.006 (0-0.019)	0.007 (0-0.019)	0.018 (0-0.047)	0.008 (0-0.021)	0.007 (0-0.021)	0.006 (0-0.019)	0.008 (0-0.023)	0.008 (0-0.022)
**FT**	0.008 (0-0.024)	0.008 (0-0.024)	*0.832 (0.781-0.884)*	0.008 (0-0.023)	0.010 (0-0.030)	0.008 (0-0.024)	0.008 (0-0.023)	0.009 (0-0.025)	0.009 (0-0.026)	0.008 (0-0.024)	0.008 (0-0.023)	0.008 (0-0.023)	0.008 (0-0.024)	0.008 (0-0.024)	0.009 (0-0.025)	0.010 (0-0.027)	0.009 (0-0.025)	0.008 (0-0.023)	0.008 (0-0.023)	0.009 (0-0.025)	0.008 (0-0.024)
**FFA**	0.008 (0-0.023)	0.008 (0-0.024)	0.008 (0-0.024)	*0.675 (0.659-0.690)*	**0.150 (0.069-0.219)**	0.008 (0-0.024)	0.008 (0-0.024)	0.008 (0-0.024)	0.009 (0-0.027)	0.008 (0-0.024)	0.008 (0-0.024)	0.008 (0-0.024)	0.008 (0-0.024)	0.008 (0-0.023)	0.008 (0-0.025)	0.019 (0-0.055)	0.010 (0-0.028)	0.008 (0-0.025)	0.008 (0-0.024)	0.015 (0-0.046)	0.009 (0-0.025)
**F32**	0.008 (0-0.024)	0.015 (0-0.037)	0.017 (0-0.044)	0.008 (0-0.024)	*0.804 (0.745-0.862)*	0.008 (0-0.024)	0.008 (0-0.024)	0.008 (0-0.024)	0.010 (0-0.030)	0.008 (0-0.022)	0.009 (0-0.025)	0.009 (0-0.026)	0.008 (0-0.024)	0.009 (0-0.025)	0.008 (0-0.024)	0.018 (0-0.051)	0.009 (0-0.025)	0.009 (0-0.026)	0.008 (0-0.024)	0.010 (0-0.030)	0.011 (0-0.031)
**FE**	0.008 (0-0.024)	0.009 (0-0.027)	0.008 (0-0.024)	0.008 (0-0.023)	**0.157 (0.108-0.206)**	*0.675 (0.660-0.690)*	0.008 (0-0.023)	0.008 (0-0.023)	0.013 (0-0.035)	0.008 (0-0.023)	0.008 (0-0.024)	0.008 (0-0.024)	0.008 (0-0.023)	0.008 (0-0.023)	0.008 (0-0.024)	0.015 (0-0.039)	0.008 (0-0.024)	0.008 (0-0.023)	0.008 (0-0.025)	0.008 (0-0.023)	0.009 (0-0.025)
**LE**	0.008 (0-0.024)	0.008 (0-0.023)	0.008 (0-0.024)	0.008 (0-0.024)	**0.166 (0.115-0.216)**	0.008 (0-0.024)	*0.675 (0.659-0.690)*	0.008 (0-0.024)	0.010 (0-0.029)	0.008 (0-0.023)	0.008 (0-0.023)	0.008 (0-0.024)	0.008 (0-0.024)	0.008 (0-0.024)	0.008 (0-0.024)	0.011 (0-0.030)	0.008 (0-0.024)	0.008 (0-0.023)	0.008 (0-0.024)	0.008 (0-0.025)	0.008 (0-0.024)
**F35**	0.008 (0-0.023)	0.008 (0-0.023)	0.008 (0-0.024)	0.008 (0-0.024)	**0.161 (0.107-0.215)**	0.008 (0-0.024)	0.008 (0-0.023)	*0.675 (0.659-0.690)*	0.013 (0-0.035)	0.008 (0-0.024)	0.008 (0-0.024)	0.008 (0-0.023)	0.008 (0-0.023)	0.008 (0-0.024)	0.008 (0-0.024)	0.013 (0-0.035)	0.008 (0-0.024)	0.008 (0-0.024)	0.008 (0-0.024)	0.008 (0-0.024)	0.008 (0-0.024)
**F29**	0.008 (0-0.023)	0.009 (0-0.027)	0.008 (0-0.024)	0.008 (0-0.024)	0.018 (0-0.049)	0.008 (0-0.023)	0.008 (0-0.024)	0.008 (0-0.022)	*0.814 (0.757-0.871)*	0.008 (0-0.024)	0.008 (0-0.024)	0.008 (0-0.025)	0.008 (0-0.023)	0.008 (0-0.023)	0.008 (0-0.024)	0.015 (0-0.043)	0.010 (0-0.031)	0.009 (0-0.025)	0.008 (0-0.023)	0.011 (0-0.031)	0.009 (0-0.026)
**FUA**	0.008 (0-0.024)	0.009 (0-0.025)	0.008 (0-0.024)	0.008 (0-0.023)	**0.096 (0.038-0.154)**	0.008 (0-0.024)	0.008 (0-0.024)	0.008 (0-0.024)	**0.041 (0.02-0.079)**	*0.675 (0.659-0.690)*	0.008 (0-0.024)	0.009 (0-0.025)	0.008 (0-0.023)	0.008 (0-0.023)	0.008 (0-0.024)	**0.048 (0-0.095)**	0.008 (0-0.024)	0.008 (0-0.024)	0.008 (0-0.024)	0.009 (0-0.026)	0.009 (0-0.025)
**F23**	0.008 (0-0.023)	0.008 (0-0.024)	0.008 (0-0.023)	0.008 (0-0.023)	**0.101 (0.018-0.183)**	0.008 (0-0.024)	0.008 (0-0.023)	0.008 (0-0.024)	0.010 (0-0.028)	0.008 (0-0.023)	*0.675 (0.659-0.690)*	0.008 (0-0.024)	0.008 (0-0.023)	0.008 (0-0.024)	0.008 (0-0.023)	**0.058 (0-0.143)**	0.011 (0-0.030)	0.008 (0-0.023)	0.008 (0-0.023)	0.024 (0-0.056)	0.010 (0-0.028)
**F11**	0.008 (0-0.023)	0.009 (0-0.027)	0.008 (0-0.024)	0.008 (0-0.023)	**0.066 (0-0.164)**	0.008 (0-0.024)	0.008 (0-0.024)	0.008 (0-0.024)	0.014 (0-0.041)	0.008 (0-0.023)	0.008 (0-0.024)	*0.680 (0.635-0.725)*	0.008 (0-0.024)	0.008 (0-0.024)	0.008 (0-0.024)	**0.099 (0-0.230)**	0.009 (0-0.027)	0.008 (0-0.024)	0.008 (0-0.024)	0.009 (0-0.026)	0.008 (0-0.024)

**RIC**	0.009 (0-0.026)	0.009 (0-0.026)	0.009 (0-0.026)	0.009 (0-0.026)	0.027 (0-0.061)	0.009 (0-0.026)	0.009 (0-0.025)	0.009 (0-0.026)	0.011 (0-0.033)	0.009 (0-0.026)	0.009 (0-0.026)	0.010 (0-0.028)	*0.676 (0.659-0.692)*	0.009 (0-0.025)	0.009 (0-0.026)	**0.130 (0.075-0.187)**	0.010 (0-0.030)	0.009 (0-0.026)	0.009 (0-0.026)	0.011 (0-0.032)	0.010 (0-0.029)
**FBG**	0.008 (0-0.023)	0.008 (0-0.024)	0.008 (0-0.024)	0.008 (0-0.024)	0.015 (0-0.045)	0.008 (0-0.024)	0.008 (0-0.024)	0.009 (0-0.024)	0.014 (0-0.037)	0.008 (0-0.024)	0.008 (0-0.023)	0.008 (0-0.024)	0.008 (0-0.024)	*0.675 (0.659-0.691)*	0.008 (0-0.024)	**0.156 (0.103-0.209)**	0.008 (0-0.024)	0.008 (0-0.024)	0.008 (0-0.024)	0.009 (0-0.025)	0.008 (0-0.024)
**F19**	0.008 (0-0.024)	0.008 (0-0.024)	0.009 (0-0.025)	0.008 (0-0.024)	0.009 (0-0.025)	0.008 (0-0.024)	0.008 (0-0.024)	0.008 (0-0.024)	0.009 (0-0.025)	0.008 (0-0.024)	0.008 (0-0.025)	0.008 (0-0.024)	0.008 (0-0.024)	0.008 (0-0.024)	*0.675 (0.659-0.690)*	**0.138 (0.079-0.197)**	0.030 (0-0.068)	0.008 (0-0.024)	0.008 (0-0.024)	0.015 (0-0.044)	0.009 (0-0.027)
**R19**	0.008 (0-0.023)	0.010 (0-0.029)	0.014 (0-0.039)	0.008 (0-0.023)	0.036 (0-0.082)	0.009 (0-0.024)	0.008 (0-0.024)	0.008 (0-0.024)	0.012 (0-0.033)	0.008 (0-0.023)	0.008 (0-0.023)	0.008 (0-0.024)	0.008 (0-0.024)	0.009 (0-0.025)	0.008 (0-0.024)	*0.768 (0.705-0.831)*	0.011 (0-0.032)	0.019 (0-0.045)	0.008 (0-0.024)	0.021 (0-0.061)	0.010 (0-0.030)
**RP**	0.008 (0-0.024)	0.008 (0-0.023)	0.008 (0-0.024)	0.008 (0-0.024)	0.013 (0-0.035)	0.008 (0-0.023)	0.008 (0-0.024)	0.008 (0-0.024)	0.009 (0-0.026)	0.008 (0-0.023)	0.008 (0-0.023)	0.008 (0-0.023)	0.008 (0-0.024)	0.008 (0-0.023)	0.008 (0-0.024)	0.015 (0-0.040)	*0.779 (0.726-0.832)*	**0.053 (0.008-0.097)**	0.008 (0-0.023)	0.009 (0-0.026)	0.009 (0-0.026)
**FP**	0.008 (0-0.023)	0.009 (0-0.025)	0.009 (0-0.027)	0.008 (0-0.023)	0.016 (0-0.040)	0.008 (0-0.023)	0.008 (0-0.024)	0.008 (0-0.024)	0.011 (0-0.032)	0.008 (0-0.024)	0.008 (0-0.023)	0.008 (0-0.024)	0.008 (0-0.023)	0.008 (0-0.024)	0.008 (0-0.023)	0.009 (0-0.026)	0.012 (0-0.035)	*0.820 (0.767-0.872)*	0.008 (0-0.024)	0.008 (0-0.024)	0.008 (0-0.025)
**RT**	0.007 (0-0.019)	0.007 (0-0.020)	0.007 (0-0.020)	0.007 (0-0.020)	0.009 (0-0.026)	0.006 (0-0.019)	0.007 (0-0.020)	0.007 (0-0.019)	0.008 (0-0.023)	0.007 (0-0.020)	0.007 (0-0.019)	0.007 (0-0.022)	0.007 (0-0.019)	0.007 (0-0.019)	0.007 (0-0.019)	**0.086 (0.033-0.140)**	0.007 (0-0.020)	0.007 (0-0.020)	*0.674 (0.660-0.688)*	**0.110 (0.049-0.172)**	0.011 (0-0.030)
**FER**	0.006 (0-0.018)	0.007 (0-0.019)	0.007 (0-0.020)	0.006 (0-0.018)	0.009 (0-0.026)	0.006 (0-0.018)	0.006 (0-0.018)	0.006 (0-0.018)	0.009 (0-0.024)	0.006 (0-0.018)	0.006 (0-0.017)	0.006 (0-0.018)	0.006 (0-0.018)	0.006 (0-0.018)	0.006 (0-0.018)	0.025 (0-0.064)	0.010 (0-0.028)	0.008 (0-0.022)	0.006 (0-0.018)	*0.724 (0.675-0.772)*	**0.129 (0.066-0.193)**
**RER**	0.006 (0-0.018)	0.009 (0-0.026)	0.007 (0-0.019)	0.007 (0-0.019)	0.014 (0-0.038)	0.006 (0-0.018)	0.006 (0-0.018)	0.006 (0-0.019)	0.030 (0-0.070)	0.006 (0-0.019)	0.006 (0-0.018)	0.006 (0-0.018)	0.006 (0-0.018)	0.006 (0-0.018)	0.006 (0-0.019)	0.027 (0-0.072)	0.009 (0-0.026)	0.011 (0-0.033)	0.006 (0-0.019)	**0.129 (0.043-0.215)**	*0.688 (0.651-0.726)*

Figure S1 A qualitative descriptor of allele frequency distribution used to infer bottlenecks in 21 smooth newt populations. This method compares allele frequency distribution to the distribution expected at mutation-drift equilibrium (L-shaped distribution).

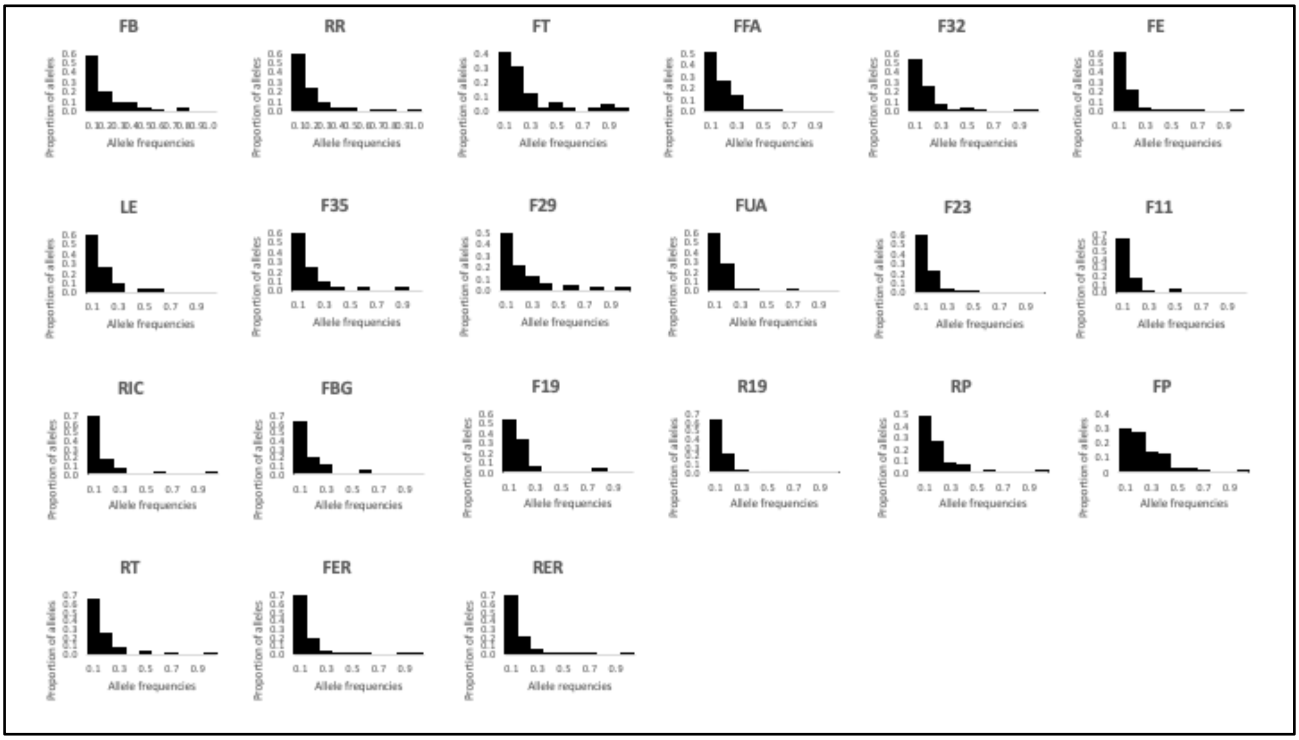

Supplement: Supplementary file 1 — Supplementary Information. [file 41598_2023_47265_MOESM1_ESM.pdf]
